# Characterization of selective and potent PI3Kδ inhibitor (PI3KD-IN-015) for B-Cell malignances

**DOI:** 10.18632/oncotarget.8702

**Published:** 2016-04-12

**Authors:** Xiaochuan Liu, Aoli Wang, Xiaofei Liang, Cheng Chen, Juanjuan Liu, Zheng Zhao, Hong Wu, Yuanxin Deng, Li Wang, Beilei Wang, Jiaxin Wu, Feiyang Liu, Stacey M. Fernandes, Sophia Adamia, Richard M. Stone, Ilene A. Galinsky, Jennifer R. Brown, James D. Griffin, Shanchun Zhang, Teckpeng Loh, Xin Zhang, Wenchao Wang, Ellen L. Weisberg, Jing Liu, Qingsong Liu

**Affiliations:** ^1^ Department of Chemistry, University of Science and Technology of China, Hefei 230036, Anhui, P. R. China; ^2^ High Magnetic Field Laboratory, Chinese Academy of Sciences, Hefei 230031, Anhui, P. R. China; ^3^ University of Science and Technology of China, Hefei 230036, Anhui, P. R. China; ^4^ CHMFL-HCMTC Target Therapy Joint Laboratory, Hefei 230031, Anhui, P. R. China; ^5^ Department of Medical Oncology, Dana Farber Cancer Institute, Harvard Medical School, Boston, MA 02115, USA; ^6^ Hefei Cosource Medicine Technology Co. LTD., Hefei 230031, Anhui, P. R. China; ^7^ Hefei Science Center, Chinese Academy of Sciences, Hefei 230031, Anhui, P. R. China

**Keywords:** PI3Kδ, leukemia, B-cell malignances, PI3K, kinase inhibitors

## Abstract

PI3Kδ is predominately expressed in leukocytes and has been found overexpressed in B-cell related malignances such as CLL and AML. We have discovered a highly selective ATP competitive PI3Kd inhibitor PI3KD-IN-015, which exhibits a high selectivity among other PI3K isoforms in both biochemical assays and cellular assay, meanwhile did not inhibit most of other protein kinases in the kinome. PI3KD-IN-015 demonstrates moderately anti-proliferation efficacies against a variety of B-cell related cancer cell lines through down-regulate the PI3K signaling significantly. It induced both apoptosis and autophagy in B-cell malignant cell lines. In addition, combination of autophagy inhibitor Bafilomycin could potentiate the moderate anti-proliferation effect of PI3KD-IN-015. PI3KD-IN-015 shows anti-proliferation efficacy against CLL and AML patient primary cells. Collectively, these results indicate that PI3KD-IN-015 may be useful drug candidate for further development of anti-B-cell related malignances therapies.

## INTRODUCTION

Mammalian PI3K lipid kinases are composed of three classes – I, II, and III, which are involved in multiple cellular functions, including signaling transduction, cell proliferation, differentiation, etc. [1] Constitutive activation or over-expressions of PI3Ks have been implicated in variety of human cancers [2]. Among these, class I PI3K lipid kinases consist of four different catalytic isoforms including PI3Kα, β, δ and γ [3]. While PI3Ka and b are ubiquitously expressed in all of the mammalian tissues, PI3Kδ and g are predominately expressed in the lymphocytes, and has been proved to be essential for B-cell survival, proliferation, and migration [4, 5]. Among these, PI3Kδ is specifically over-expressed/ aberrantly activated in a variety of B-cell malignances such as CLL and AML [6, 7]. Therefore, PI3Kδ has been extensively studied as a therapeutic target in hematologic malignancies. Currently there are several PI3Kδ inhibitors in different stages of clinical development including AMG319 (phase I), GSK2269557 (Phase II), INCB040093 (phase I), TGR-1202 (phase I), UCB-5857 (phase I) and CAL-101 (Idelalisib, approved [8]) which is the first selective PI3Kδ inhibitor approved by US Food and Drug Administration (FDA) for the treatment of chronic lymphocytic leukemia (CLL) and B-cell non-Hodgkin lymphoma(FL) and relapsed small lymphocytic lymphoma(SLL) [9–11]. Here we report the discovery of another potent and selective PI3Kδ inhibitor, PI3KD-IN-015, which exhibits potent and selective inhibitory effects against the PI3Kδ-mediated signaling pathway and the proliferation of both established cancer cell lines and primary CLL and AML patient cells.

## RESULTS

### PI3KD-IN-015 is a potent and selective inhibitor of PI3Kδ

**Figure 1:**
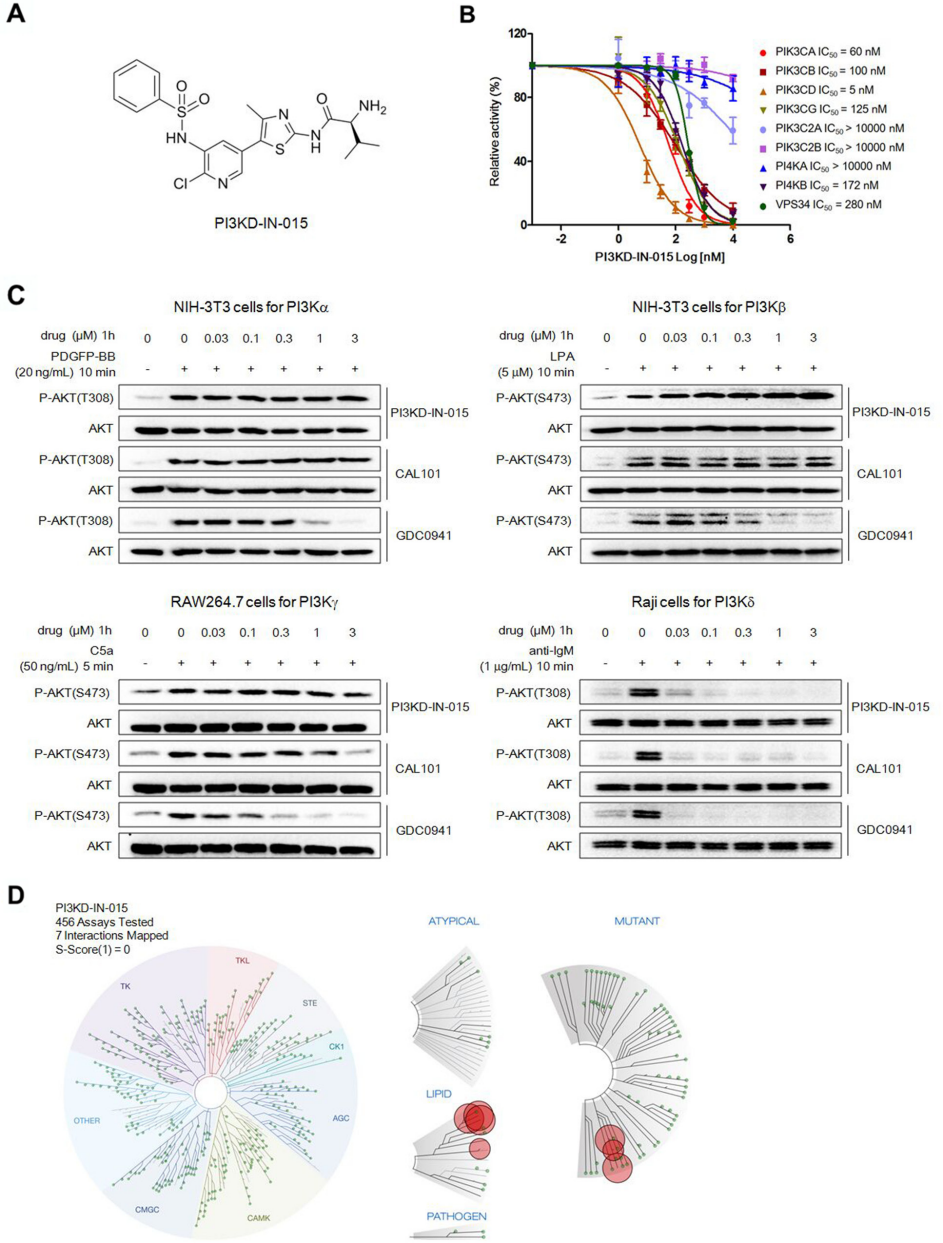
Discovery of PI3KD-IN-015 as a potent and selective PI3Kδ inhibitor **A.** Chemical Structure of PI3KD-IN-015. **B.** ADP-glo biochemical assay determination of IC50 of PI3KD-IB-015 against different PI3K isoforms. **C.** Determination of PI3KD-IN-015 inhibitory activities against PI3Kα, β, δ and γ in cellular background. **D.** TreeSpot™ demonstration of PI3KD-IN-015 selectivity against a panel of 456 kinases with DiscoveRx KinomeScan™ technology with an S (1) score =0.

Starting from an aminothiazole scaffold, with the structure guided drug design approach, the focused medicinal chemistry efforts lead us to identify a PI3Kδ inhibitor PI3KD-IN-015 (Chemical structure shown in Figure 1A). The ADP-glo biochemical assay with purified enzymes demonstrated that among class I PI3K kinases PI3KD-IN-015 inhibited PI3Kδ with a IC50 of 5 nM, meanwhile it exhibited 12 fold selectivity against PI3Kβ PI3Kα (IC50: 60 nM), 20 fold selectivity against PI3Kβ (IC50: 100 nM), and 25-fold selectivity against PI3Kγ (IC50: 125 nM). It did not show apparent activity against class II PI3Ks PIK3C2A and PIK3C2B (IC50 over 10mM) and showed 56-fold selectivity against Class III kinase Vps34 (IC50: 280 nM). In addition, PI3KD-IN-015 did not inhibit PI4KA (IC50 over 10mM) and exhibited 34-fold selectivity against PI4KB (IC50: 172 nM). (Figure 1B) Its selectivity profile was better than pan-PI3K inhibitor GDC-0941 but less than well-established PI3Kδ inhibitor CAL-101 in general. (Table 1) However, it displayed better selectivity profile than CAL-101 between PI3Kδ and PI3Kγ. In order to further confirm the selectivity of PI3KD-IN-015 among the class I PI3Ks, we then looked at the cellular effects of PI3K-IN-015 on PI3K signaling pathway. The catalytic activity of PI3Kδ triggers the signal transduction cascades mediated by Akt, and the phosphorylation of Thr-308 residue on the activation loop of Akt kinase domain is often used as a marker for Akt activation. After each PI3K isoform is individually activated by different stimulating reagents, addition of PI3K-IN-015 specifically inhibited PI3Kδ-mediated Akt phosphorylation at T308 residue inRaji cells stimulated by anti-IgM with IC50 at 13 nM, but it did not showed apparent inhibitory effect on Akt activation controlled by the other three class I PI3K isoforms (α, β and γ: EC50 over 3mM). (Figure 1C and Table 1) The well-established PI3Kδ specific inhibitor, CAL-101, showed similar effects as PI3KD-IN-015 with an EC50 of 2.3 nM against PI3Kδ and over 1000-fold less potent against the other three isoforms. While the pan-PI3K inhibitor, GDC-0941, inhibited Akt phosphorylation induced by all four PI3K isoforms with submicromolar IC50s though it also exhibited the relative selectivity to PI3Kδ (EC50 of 4.3 nM over 624 nM, 176 nM and 129 nM against α, β and γ isoform) [12]. (Figure 1C and Table 2)

**Table 1:** Comparison of PI3KD-IN-015, CAL-101 and GDC-0941 biochemical IC50s among Class I PI3K isoforms

Drug/PI3K isoforms biochemical IC50(nM)	Alpha	Beta	Gamma	Delta
PI3KD-IN-015	60	100	125	5
CAL101	1089	664	25	7
GDC0941	22	137	40	12

**Table 2:** Efficacy of PI3K inhibitors against PI3K isoforms activity in cellular background^a^

Drug/PI3K isoforms cellular EC50(nM)	Alpha	Beta	Gamma	Delta
PI3KD-IN-015	>3000	>3000	>3000	13
CAL101	>3000	>3000	2324	2.3
GDC0941	624	176	129	4.3

a all EC50s were obtained by triplet testing.

To further characterize the selectivity profile of PI3KD-IN-015 in the kinome wide range, we next performed kinome selectivity analysis on PI3KD-IN-015 using DiscoveRx's KinomeScan™ technology [13]. PI3KD-IN-015 did not target any other protein kinases except for a few lipid kinases, including isoforms and mutants of PI3K and PI4K at a concentration of 1mM. (Figure 1D and Supplementary Table S1) Collectively, these results indicated that PI3KD-IN-015 was a potent and selective PI3Kδ inhibitor.

### Structural basis for PI3KD-IN-015's selectivity among PI3K isoforms

**Figure 2:**
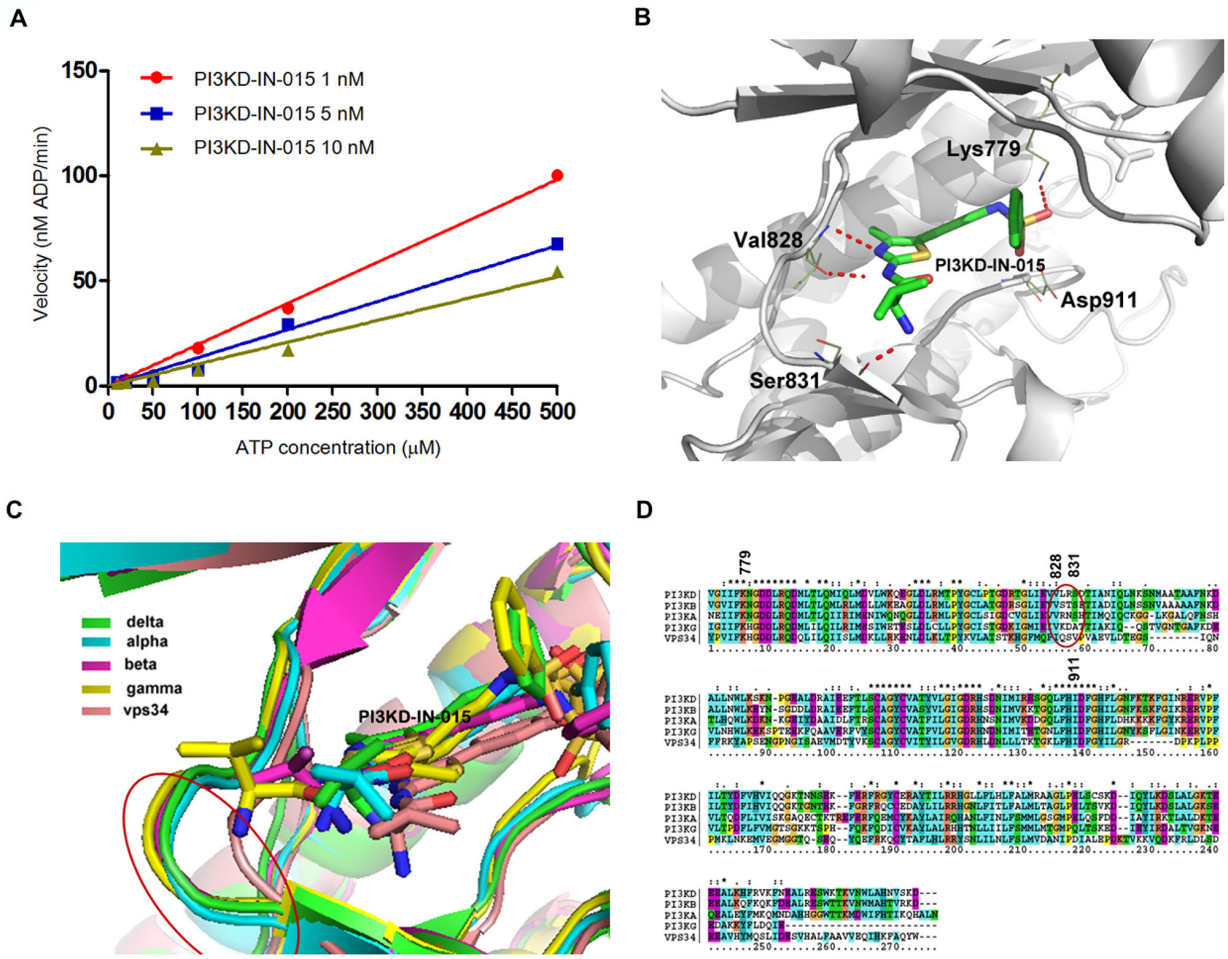
Structural basis for PI3KD-IN-015's selectivity among PI3K isoforms **A.** ATP competitive kinetic study of PI3KD-IN-015 against PI3Kδ. **B.** PI3KD-IN-015 was docked into PI3Kδ (homology model, template PDB ID: 2WXF). **C.** Superimposition of PI3KD-IN-015 in complex with PI3Kα, β, δ, γ and vps34. **D.** Sequence alignment of PI3Kα, β, δ, γ and vps34 kinase domain.

In order to further understand the binding mode of PI3KD-IN-015 with PI3Kδ, we first studied the binding kinetics of it. The *in vitro* ATP-competitive assay on PI3Kδ demonstrated that PI3KD-IN-015 was an ATP competitive inhibitor. (Figure 2A) We then docked the inhibitor into PI3Kδ homology-model structure (template: PDB ID: 2WXF) and the result showed that PI3KD-IN-015 formed two hydrogen bonds in the hinge binding area with Val828 via N- and NH- on the aminothiazole scaffold. (Figure 2B) The NH2- on Valine moiety of PI3KD-IN-015 formed a hydrogen bond with Ser831 near the hinge binding area. In addition, the O- on the sulfonamide moiety formed a hydrogen bond with Lys779 in the inner hydrophobic pocket. To better understand the selectivity among the different class I PI3K isoforms, we further docked PI3KD-IN-015 into structures of PI3Kα (PDB ID:4JPS), β(homology-model, template: PDB ID:2Y3A) γ(PDB ID:4WWO) and VPS34 (PDB: ID3LS8) individually and superimposed them together. (Figure 2C) Detailed analysis of those four isoforms’ highly similar sequences, we found that in the hinge binding area (828-832 corresponding to PI3Kδ) the amino acids residues exhibited difference and this may explain the selectivity among these isoforms. (Figure 2D)

### PI3KD-IN-015 blocks PI3Kδ mediated downstream signaling pathways

**Figure 3:**
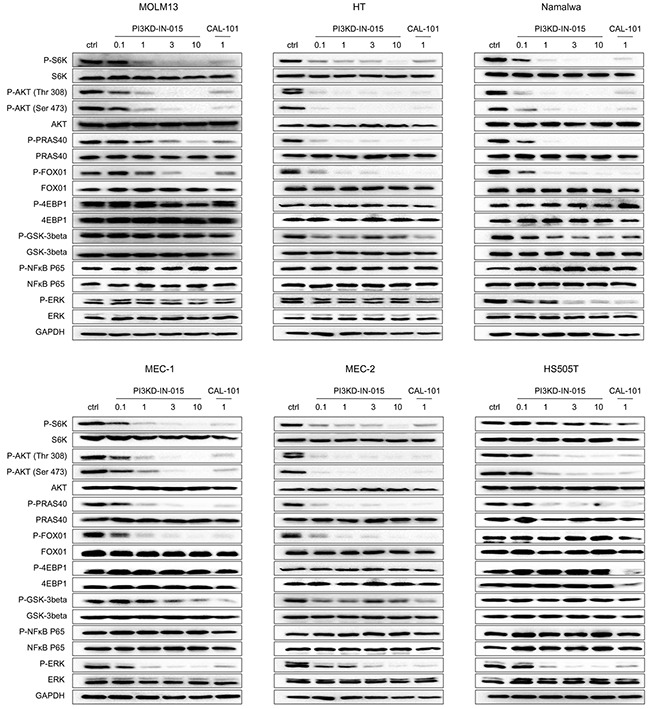
PI3KD-IN-015 effect on PI3K related signaling pathways in MOLM13 (AML), HT(B-NHL), MEC-1(CLL), MEC-2 (CLL) and HS505T(CLL)

We next investigated the effects of PI3KD-IN-015 on PI3K mediated downstream signaling in various B-cell malignant cell lines. The results showed that PI3KD-IN-015 potently inhibited the phosphorylation of Akt at both Thr308 and Ser473 sites, the downstream target of PI3K, among MOLM-13 (AML), HT (B-NHL), Namalwa (Burkitt Lymphoma), MEC-1 (CLL), MEC-2 (CLL), and HS505T (CLL) cells, with EC50 less than 1 μM (Figure 3). In addition, the substrates of Akt, such as PRAS40 GSK3b and FOXO1, also exhibited decreased phosphorylation. The AKT downstream signaling mediator mTOR's substrates S6K phosphorylation was also significantly decreased at 1mM in MOLM13, HT, Namalwa, MEC-1, MEC-2 cells but not in HS505T cells. Interestingly, the other well established mTOR's substrate 4EBP1's phosphorylation was not changed at all in all of the cell lines which further confirmed that PI3KD-IN-015 has no direct inhibitory effect against mTOR kinase though it is structurally very similar to PI3Ks. In addition, NF-kB P65 phosphorylation was not inhibited in any of these tested cell lines with PI3KD-IN-015 treatment. Erk phosphorylation was reduced upon PI3KD-IN-015 treatment in Namalwa, MEC-1, MEC-2 and HS505T cells but not in MOLM13 and HT, as what CAL-101 did. Collectively these data illustrated that PI3KD-IN-015 is a potent PI3Kδ inhibitor in cellular context, as what CAL-101 did, but their pharmacological profile may be slightly different.

### PI3KD-IN-015 exhibits anti-proliferative activity against B-cell related cancer cell lines

We next screened PI3KD-IN-015 against a panel of cancer cell lines derived from B-cell malignancies. The results showed that PI3KD-IN-015 exhibited moderate anti-proliferative effects against most of the cell lines including AML, B-cell lymphoma and multiple myeloma cell lines with GI50 between 1-10 μM but did not exhibit apparent inhibitory activity against CLL cell lines. (Table 3) CAL-101 did not exhibit apparent inhibitory activity against most of the cell lines tested except the AML cell line OCI-AML-3, MOLM14 and mantle cell lymphoma cell REC-1. In contrast, the pan-PI3K inhibitor GDC-0941 significantly inhibited the cell growth in most of the cell lines. However, none of the three inhibitors, PI3KD-IN-015, CAL-101 and GDC-0941, displayed anti-proliferative effects against three CLL cell lines tested (HS505T, MEC-1 and MEC-2), This is consistent with the previous report that PI3Kδ inhibitors do not show strong direct inhibition on cell growth, but rather alter microenvironment and indirectly inhibit cell proliferation in CLL patients.[14]

**Table 3:** PI3KD-IN-015, CAL-101 and GDC-0941 anti-proliferative effect against a panel of B-cell malignances related cancer cell lines^a^

Cell lines	Cell type	PI3KD-IN-015 GI50 (μM)	CAL-101 GI50 (μM)	GDC-0941 GI50 (μM)
PF382	ALL	3.2	>10	0.46
MV4-11	AML	1.9	>10	1.4
U937	AML	5.1	>10	1.6
CMK	AML	10-3	>10	0.3
NB4	AML-3	10-3	>10	1.0
HL-60	AML-M2	2.3	>10	0.16
OCI-AML-3	AML-M4	3.1	2.4	0.73
OCI-AML-2	AML-M4	1.8	>10	2
NOMO-1	AML-M5	1.4	>10	1
MOLM-14	AML-M5	2.2	7.8	0.3
MOLM-13	AML-M5	0.94	>10	0.15
NALM-6	AML-M5	1.8	>10	0.39
SKM-1	AML-M5	2.6	>10	0.54
HEL	AML-M6	>10	>10	>10
HT	B-cell lymphoma	0.86	>10	0.5
Namalwa	Burrkit lymphoma	1.4	>10	0.39
Ramos(new)	Burrkit lymphoma	2	>10	2.1
Hs 505T	CLL	>10	>10	>10
MEC-1	CLL	>10	>10	>10
MEC-2	CLL	>10	>10	>10
JVM-2	Mantle cell lymphoma	>10	>10	>10
RPMI8226	MM	2.5	>10	2.6
AMO-1	MM	1.1	>10	0.3

a all GI50s were obtained by triplet testing.

To further examine the anti-growth efficacy of PI3KD-IN-015, we then performed the clonogenic assays using HT (B-NHL), MEC-1 (CLL) and MOLM13 (AML). Interestingly, PI3KD-IN-015 displayed strong anti-colony formation activity in all of the 3 cell lines (HT: EC50: 374 nM), MEC-1: EC50: 765 nM and MOLM13: EC50: 215 nM). (Supplememtary Figure S1) This is interesting that since for the CLL cell MEC-1, PI3KD-IN-015 has no direct effect on its proliferation (over 10 μM, Table 2) but can strongly inhibit the colony formation. This may further confirm the clinically observed results from CAL-101 that PI3Kδ inhibitor has little direct cytotoxic effect against CLL cells but may indirectly inhibit its proliferation through the microenvironment [14].

### PI3KD-IN-015 induces apoptosis and autophagy in B-cell related cancer cell lines

**Figure 4:**
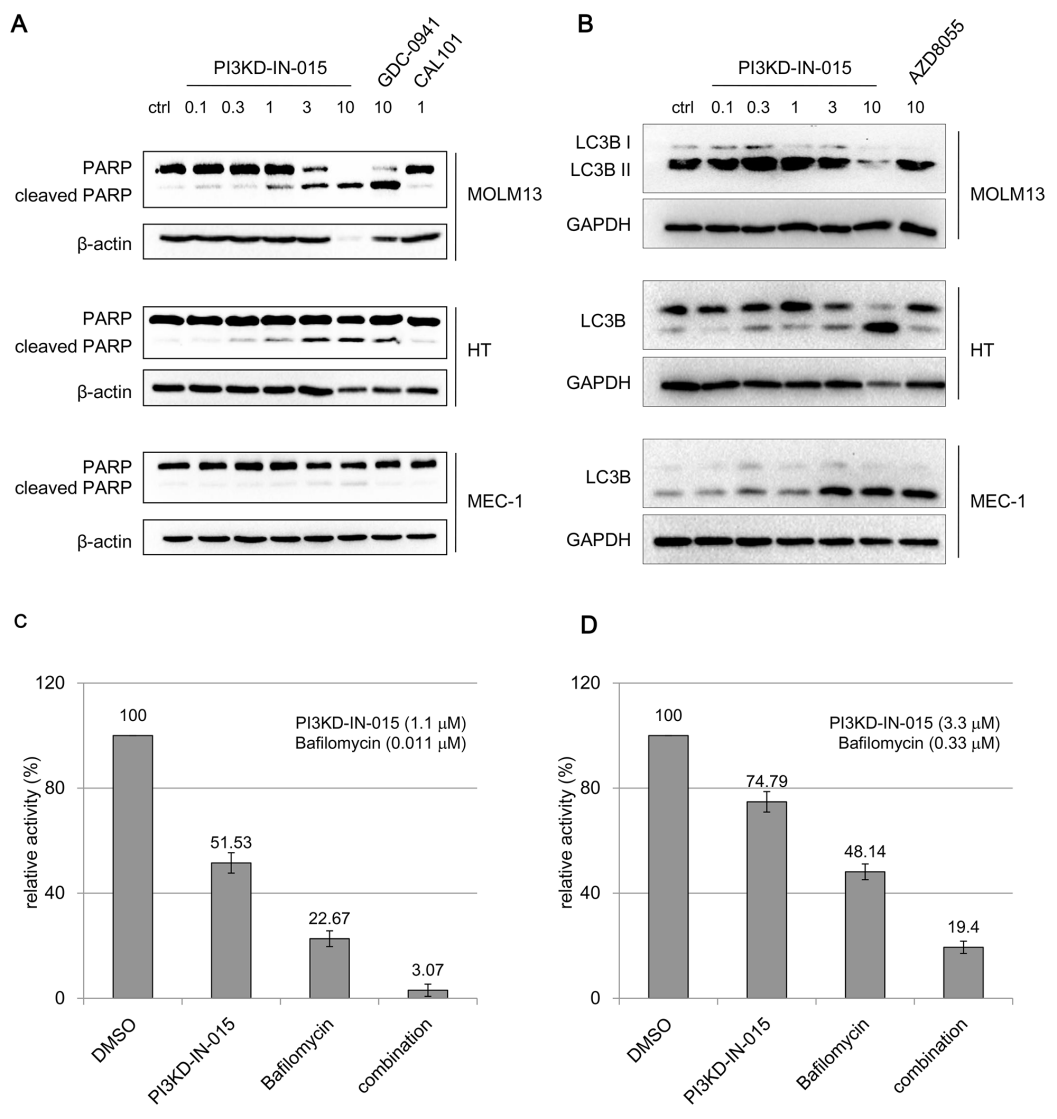
PI3KD-IN-015 effect of apoptosis and autophagy in AML, B-NHL and CLL cells **A.** PI3KD-IN-015 induction of apoptosis in MOLM13 (AML) and HT (B-NHL) and MEC-01(CLL) cells. **B.** PI3KD-IN-015 induction of autophagy in MEC-01(CLL), HT (B-NHL) but not in MOLM13(AML) cells. **C.** PI3KD-IN-015 exhibit greater anti-proliferative efficacy against HT and MEC-01 cells by combination of autophagy inhibitors Bafilomycin.

PI3KD-IN-015 induced apparent PARP cleavage in MOLM-13 cell (AML), HT cell (B-NHL), but only weakly in MEC-1 cell (CLL) within 12 hours. (Figure 4A) Similar trend was observed for the reported pan-PI3K inhibitor GDC-0941. Because of the close involvement of PI3K/Atk/mTOR pathway in autophagy, we also examined whether PI3KD-IN-015 has any effect in autophagy induction. In MOLM-13 cell, which has been observed for obvious apoptosis, no apparent autophagy induction was observed. (Figure 4B) While in both HT cells and MEC-1 cells, treatment of PI3KD-IN-015 significantly increased the production of LC3B II, indicating that PI3KD-IN-015 can induce autophagy, probably through its activity against PI3Kδ. As a positive control, mTOR inhibitor, AZD8055 also showed a similar autophagy inducing effect. Given the fact that autophagy is often used by cancer cells as a protective mechanism, we then tried to combine PI3KD-IN-015 with autophagy inhibitor Bafilomycin in HT cells and MEC-1 cells. The result showed that Bafilomycin could synergistically potentiate PI3KD-IN-015's anti-proliferative efficacy in HT and MEC-1 cells. (Figure 4C, 4D)

### PI3KD-IN-015 inhibits the proliferation of primary CLL and AML patient cells

We then investigated PI3KD-IN-015's effect on patient primary cells. The result showed that, PI3KD-IN-015 effectively suppressed the growth of 2 of 3 CLL primary cells and was effective against most of AML patient cells with GI50 less than 10 μM (Table 4 and Supplementary Table S2). CAL-101 did not show anti-proliferative effect to most of the patient samples. Again this indicated that PI3KD-IN-015 might bear different pharmacological profile with CAL-101.

**Table 4:** PI3KD-IN-015 and CAL-101 anti-proliferative efficacy against CLL and AML patient primary cell^a^

GI_50_(μM)	CAL-101	PI3KD-IN-015
CLL16	>10	7.8
CLL19	>10	1.5
CLL18	>10	>10
AML1	>10	1.83
AML2	1.01	0.85
AML3	>10	9.2
AML4	>10	2.8
AML5	>10	6.3
AML6	>10	>10

a all GI50s were obtained by triplet testing.

## DISCUSSION

The seminal discovery of the highly selective PI3Kδ inhibitor CAL-101 (Idelalisib) has opened a new scenario in the CLL clinical treatment. However, both the preclinical and clinical observation have confirmed that unlike other target therapies, which will show direct cytotoxicity against the cancer cell itself directly, inhibition of PI3Kδ itself only exhibits limited cytotoxicity against the cancer cells. Rather it majorly exert its antagonizing cell survival efficacy through interfering the microenvironment, such as by blocking the production of cytokines, including TNF-a, IL-6, etc, that will prevent the leukemia cells from circulating back to the lymph nodes and bone marrow for the further proliferation [14]. In order to improve the clinical efficacy, combination of other chemotherapies and signaling pathway inhibitors have been suggested and tested in the clinical trials [15]. Here both our newly discovered selective PI3Kδ inhibitor PI3KD-IN-015 and CAL-101 proved again that in the CLL cell lines they did not exhibit any apparent anti-proliferative efficacies *in vitro*. Interestingly, we found that treatment of CLL cell lines induced autophagy, which might provide a pro-survival mechanism for overcoming the cytotoxicity effect of the compound. The roles of autophagy in hematologic malignancies are still controversial, as it may promote either cell survival or cell death. The enhanced anti-proliferative efficacy by combination of autophagy inhibitor Bafilomycin has provided an alternative approach to improve the anti-leukemic efficacy of PI3Kδ inhibitors.

Interestingly, despite of the overexpression of PI3Kδ has been reported in the AML and B-NHL, CAL-101 did not show too much efficacy against most of these cell lines in our testing. But, the PI3KD-IN-015 has displayed moderate anti-proliferative activities against most of the AML, MM, and B-cell lymphoma cell lines indicating that these two drugs may bear different pharmacological profiles besides the PI3Kδ.

In summary, we report here the discovery of a new highly potent and selective PI3Kδ inhibitor PI3KD-IN-015 that potently inhibits PI3Kδ mediated signaling pathway, induces apoptosis and autophagy in AML/CLL cell lines and inhibits the proliferation of CLL and AML patient primary cells. PI3KD-IN-015 might serve as a supplementary to the armory to fight the PI3Kδ mediated B-cell malignances.

## MATERIALS AND METHODS

### Chemical reagents

PI3KD-IN-015 was synthesized in the lab with the procedure provided in the Supplementary Materials section. CAL-101, GDC-0941 and AZD8055 was purchased from Haoyuan Chemexpress Inc.(Shanghai, China)

### Cell lines and cell cultures

The human cancer cell lines MV4-11, SKM-1, SU-DHL-2, U2932, JVM-2, Namalwa were purchased from the American Type Culture Collection (ATCC) (Manassas, VA, USA). The human AML cell lines MOLM-13, MOLM14, HEL and the human ALL cell line PF382 were provided by Dr. Scott Armstrong, Dana Farber Cancer Institute (DFCI), Boston, MA. The AML line, NB4 (KRAS A18D), was obtained from Dr. Gary Gilliland. ALL the rest cell lines were purchased from Cobioer Biosciences CO. LTD (NanJing, China). MV4-11 and MEC-1 was cultured in IMDM media (Corning, USA) with 10% FBS and supplemented with 2% L-glutamine and 1% pen/strep. OCI-AML-3 was cultured in α-MEM media (Cornig, USA) with 10% FBS and supplemented with 2% L-glutamine and 1% pen/strep. OCI-AML-2 was cultured in α-MEM media (Cornig, USA) with 20% FBS and supplemented with 2% L-glutamine and 1% pen/strep. ALL the rest cell lines were cultured in RPMI 1640 media (Corning, USA) with 10% fetal bovine serum (FBS) and and supplemented with 2% L-glutamine 1% penicillin/streptomycin. All cell lines were maintained in culture media at 37°C with 5% CO2.

### Primary cells

Mononuclear cells were isolated from AML patients. Mononuclear cells were isolated by density gradient centrifugation through Ficoll-Plaque Plus (Amersham Pharmacia Biotech AB, Uppsala, Sweden) at 2000 rpm for 30 minutes, followed by two washes in 1X PBS. Freeze-thawed cells were then cultured in liquid culture (DMEM, supplemented with 20% FBS). All blood and bone marrow samples from AML patients were obtained through written consent under approval of the Dana Farber Cancer Institute Institutional Review Board. The ethics committees approved the consent procedure.

Peripheral blood mononuclear cells (PBMCs) from individuals with CLL were isolated by density centrifugation through Ficoll and frozen for each subject. Those subjects with low white counts whose CLL cell purity was expected to be < 85% underwent B cell isolation using RosetteSep. The protocol was approved by the Dana-Farber Harvard Cancer Center Institutional Review Board and all subjects signed written informed consent prior to participation.

### Antibodies

The following antibodies were purchased from Cell Signaling Technology (Danvers, MA): Akt (pan) (C67E7) Rabbit mAb (#4691), Phospho-Akt (Thr308) (244F9) Rabbit mAb (#4056), Phospho-Akt (Ser473) (D9E) XP® Rabbit mAb (#4060), GAPDH (D16H11) XP® Rabbit mAb, GSK-3β (27C10) Rabbit mAb (#9315), Phospho-GSK-3β (Ser9) (5B3) Rabbit mAb (#9323), FoxO1 (C29H4) Rabbit mAb (#2880), Phospho-FoxO1 (Thr24)/FoxO3a (Thr32)/FoxO4 (Thr28) (4G6) Rabbit mAb (#2599), PRAS40 (D23C7) XP® Rabbit mAb (#2691), Phospho-PRAS40 (Thr246) (C77D7) Rabbit mAb (#2997), 4E-BP1 (#9644), 4E-BP1 (53H11) Rabbit mAb (#9644), p70 S6 Kinase (49D7) Rabbit mAb (#2708), Phospho-p70 S6 Kinase (Thr389) (108D2) Rabbit mAb (#9234). SQSTM1/p62 (D5E2) Rabbit mAb (#8025), LC3B (D11) XP® Rabbit mAb (Biotinylated) (#12513). PARP (46D11) rabbit mAb (#9532), Caspase-3 (8G10) rabbit mAb (#9665), Phospho-p44/42 MAPK (Erk1/2) (Thr202/Tyr204) (197G2) Rabbit mAb (#4377), p44/42 MAPK (Erk1/2) (137F5) Rabbit mAb (#4695), Phospho-NF-ΚB P65 (Ser 536) (93H1) Rabbit mAb (#3033), NF-ΚB P65 (C22B4) Rabbit mAb (#4764). Protein lysate preparation and antibodies were used for immunoblotting. Lysophosphatidic Acid (#sc-201053) was purchased from SantaCruze, Recombinant Human PDGF-BB (#220-BB) was purchased from R&D, Recombinant Human C5a was purchased from Peprotech (#300-70), goat anti-IgM (#5C07615) was purchased from Meridian, these factors were used for PI3K isoforms cellular selectivity assay.

### PI3KD-IN-015 docking

The PDB structures for different PI3K isoforms(α, γ and vps34) were downloaded from Protein Data Bank, PDB ID 4JPS for PI3Kalpha, 4WWO for gamma, and 3LS8 for vps34. The 3D structure of PI3Kβ was not released, here we obtained by homology model using PDB id 2Y3A as template with modeler software, in the same method, PI3Kδ homology model using 2WXF as template by using modeler software. Then the 3D-structure alignment was carried out by using strap tool and the sequence alignment was carried out by using cluster 2.1. All PI3KD-IN-015 were docked to corresponding isoforms by using PLANTS docking software with the default parameters.

### PI3Ks isoform ADP-glo assay

The ADP-Glo™ kinase assay (Promega, Madison, WI) was used to screen PI3KD-IN-015 for its PI kinases inhibition effects. Kinase reaction system contains 4.95 μL PIK3CA (0.16 μg/mL), PIK3CB (6 μg/mL), PIK3CD (2.2 μg/mL) or PIK3CG (12 μg/mL) (Invitrogen, USA), 0.55 μL of serially diluted PI3KD-IN-015, and 5.5 μL substrate PIP2:PS (0.1mM) (Invitrogen, USA) with 10 μM ATP (Promega, Madison, WI). The kinase reaction system contains 4.95 μL PIK3CD (2.2 μg/mL), PIK3CG (12 μg/mL) (Invitrogen, USA), 0.55 μL of serially diluted PI3KD-IN-015, and 5.5 μL substrate PIP2:PS (0.1mM) (Invitrogen, USA) with 50 μM ATP (Promega, Madison, WI). The kinase reaction system contains 4.95 μL PIK3C2A (13 μg/mL), PIK3C2B (20 μg/mL) (Invitrogen, USA), 0.55 μL of serially diluted PI3KD-IN-015, and 5.5 μL substrate PI (0.1mM) (Invitrogen, USA) with 50 μM ATP (Promega, Madison, WI). The kinase reaction system contains 4.95 μL PI4KA (13 μg/mL), PI4KB (2.2 μg/mL) or VPS34 (2.2 μg/mL) (Invitrogen, USA), 0.55 μL of serially diluted PI3KD-IN-015, and 5.5 μL substrate PI:PS (0.1mM) (Invitrogen, USA) with 50 μM ATP (Promega, Madison, WI). The reaction in each tube was started immediately by adding ATP and kept going for an hour under 37°C. After the tube cooled for 5 minutes at room temperature, 5 μL solvent reactions were carried out in a 384-well plate. Then 5 μL of ADP-Glo™ reagent was added into each well to stop the reaction and consume the remaining ADP within 40 minutes. At the end, 10 μL of kinase detection reagent was added into the well and incubated for 30 minutes to produce a luminescence signal. Luminescence signal was measured with an automated plate reader (Perkin-Elmer Envision) and each measurement was performed in triplicate.

### Kinase kinetic assay

Kinetic analyses of PI3KCD were performed using a luminometric kinase assay varying the concentration of ATP using the ADP-Glo reagents (Promega). The serially diluted PI3KD-IN-015 and PI3KCD (2.2 μg/mL) were assayed in a reaction (10 μL) containing 50 mM HEPES (pH 7.5), 3 mM MgCl_2_, 1 mM EGTA, 100 mM NaCl_2_, 0.03% CHAPS. After 60 min incubation at RT, varied concentrations of ATP, 0.1mM substrate PIP2:PS were added and incubated for 60 min at 37°C. The overall rate of reaction was determined as the slope of the decreasing phase of the reaction. Each data point was collected in duplicate and kinetic parameters were obtained using Prism 5.0 (GraphPad Software, San Diego, CA).

### PI3Ks isoform in cell EC50 assay

Raji cell was cultured in 10% FBS-containing RPMI and NIH-3T3, RAW264.7 macrophages cells were cultured in 10% FBS-containing DMEM medium. For selectivity against PI3K α, β, γ, δ isoforms, NIH-3T3, NIH-3T3, RAW264.7 macrophages and Raji cells were seeded in a 6-well tissue culture plate and starved for 24 hours, then incubated with compounds at the desired concentrations for 1 hour followed by 20ng/ml PDGF-BB for 10min, 5μM LPA for 10min, 50ng/ml c5a for 5min, 1μg/ml anti-IgM for 10min. Cells were lysed and AKT phosphorylation was determined by Western Blotting. Intensity of the bands was determined using ImageJ 1.42q (NIH, USA) and normalized to total AKT (loading control).

### Anti-proliferative assay

Cells were grown in 96-well culture plates (3000-4000/well). The compounds of various concentrations were added into the plates, DMSO concentrations were kept constant and did not exceed 0.1% of the total volume. Cell proliferation was determined after treatment with compounds for 72 hours. Cell viability was measured using the CellTiter–Glo Assay Kit (Promega, USA), according to the manufacturer's instructions, and luminescence was measured in a multi-label reader (Envision, PerkinElmer, USA). Data were normalized to control groups (DMSO) and represented by the mean of three independent measurements with standard error <20%. GI_50_ values were calculated using Prism 5.0 (GraphPad Software, San Diego, CA).

### Colony formation assay

In brief, 1 mL of 3 % agarose combined with 1 mL HT, REC-1, MEC-1 and MOLM-13 growth media was used as the bottom agar in a 6-well plate. 1000 cells in 1.8 mL growth media was combined with 0.2 mL of 3% agarose solution and 2 μL serially diluted PI3KD-IN-015, then plated on top of the bottom layer. Cells were maintained in a humidified 5% CO_2_ incubator at 37°C for 15 days. On the 15th day, the numbers of colonies in each well were counted and each measurement was performed in triplicate.

### Signaling pathway effect examination

MOLM13, HT, MEC-1, MEC-2 cells were cultured in 10% FBS-containing RPMI and HS505T cells were cultured in 10% FBS-containing DMEM medium. The serially diluted PI3KD-IN-015 was added to cells for 1 hour. The cells were collected and lysed, and cell lysates were analyzed by western blotting.

### Apoptosis

MOLM13, HT, MEC-1 cells were cultured in 10% FBS-containing RPMI medium. The serially diluted PI3KD-IN-015 was added to cells for 12 hours. Then, apoptosis of MOLM13, HT, MEC-1 cells were detected by western-blot using PARP, GAPDH antibody (CST).

### Autophagy

MOLM13, HT, MEC-1 cells were treated with serially diluted PI3KD-IN-015 for 12 hours. Cells were then washed in PBS and lysed in cell lysis buffer and detected by western-blot using LC3B, GAPDH antibody (Cell signaling Technology).

### Combination study

The cell lines HT and MEC-1 were grown in 96-well culture plates (4000/well), respectively. Combination of autophagy inhibitor Bafilomycin with PI3KD-IN-015 treated cell lines. Cell proliferation was determined after treatment with compounds for 72 hours. Cell viability was measured using the CellTiter–Glo assay (Promega, USA), data were normalized to control groups (DMSO) and represented by the mean of three independent measurements with standard error <20%.

## SUPPLEMENTARY INFORMATION, FIGURES AND TABLES





## References

[1] Vanhaesebroeck B, Guillermet-Guibert J, Graupera M, Bilanges B (2010). The emerging mechanisms of isoform-specific PI3K signalling. Nat Rev Mol Cell Biol.

[2] Fruman DA, Rommel C (2014). PI3K and cancer: lessons, challenges and opportunities. Nat Rev Drug Discov.

[3] Reif K, Okkenhaug K, Sasaki T, Penninger JM, Vanhaesebroeck B, Cyster JG (2004). Cutting edge: differential roles for phosphoinositide 3-kinases, p110gamma and p110delta, in lymphocyte chemotaxis and homing. J Immunol.

[4] Bilancio A, Okkenhaug K, Camps M, Emery JL, Ruckle T, Rommel C, Vanhaesebroeck B (2006). Key role of the p110delta isoform of PI3K in B-cell antigen and IL-4 receptor signaling: comparative analysis of genetic and pharmacologic interference with p110delta function in B cells. Blood.

[5] Herman SEM, Gordon AL, Wagner AJ, Heerema NA, Zhao WQ, Flynn JM, Jones J, Andritsos L, Puri KD, Lannutti BJ, Giese NA, Zhang XL, Wei L, Byrd JC, Johnson AJ (2010). Phosphatidylinositol 3-kinase-delta inhibitor CAL-101 shows promising preclinical activity in chronic lymphocytic leukemia by antagonizing intrinsic and extrinsic cellular survival signals. Blood.

[6] Fruman DA, Rommel C (2011). PI3Kdelta inhibitors in cancer: rationale and serendipity merge in the clinic. Cancer Discov.

[7] Billottet C, Grandage VL, Gale RE, Quattropani A, Rommel C, Vanhaesebroeck B, Khwaja A (2006). A selective inhibitor of the p110delta isoform of PI 3-kinase inhibits AML cell proliferation and survival and increases the cytotoxic effects of VP16. Oncogene.

[8] Stark AK, Sriskantharajah S, Hessel EM, Okkenhaug K (2015). PI3K inhibitors in inflammation, autoimmunity and cancer. Curr Opin Pharmacol.

[9] Brown JR, Byrd JC, Coutre SE, Benson DM, Flinn IW, Wagner-Johnston ND, Spurgeon SE, Kahl BS, Bello C, Webb HK, Johnson DM, Peterman S, Li D, Jahn TM, Lannutti BJ, Ulrich RG (2014). Idelalisib, an inhibitor of phosphatidylinositol 3-kinase p110delta, for relapsed/refractory chronic lymphocytic leukemia. Blood.

[10] Gopal AK, Kahl BS, de Vos S, Wagner-Johnston ND, Schuster SJ, Jurczak WJ, Flinn IW, Flowers CR, Martin P, Viardot A, Blum KA, Goy AH, Davies AJ, Zinzani PL, Dreyling M, Johnson D (2014). PI3Kdelta inhibition by idelalisib in patients with relapsed indolent lymphoma. N Engl J Med.

[11] Fruman DA, Cantley LC (2014). Idelalisib--a PI3Kdelta inhibitor for B-cell cancers. N Engl J Med.

[12] Raynaud FI, Eccles SA, Patel S, Alix S, Box G, Chuckowree I, Folkes A, Gowan S, De Haven Brandon A, Di Stefano F, Hayes A, Henley AT, Lensun L, Pergl-Wilson G, Robson A, Saghir N (2009). Biological properties of potent inhibitors of class I phosphatidylinositide 3-kinases: from PI-103 through PI-540, PI-620 to the oral agent GDC-0941. Molecular cancer therapeutics.

[13] Fabian MA, Biggs WH, Treiber DK, Atteridge CE, Azimioara MD, Benedetti MG, Carter TA, Ciceri P, Edeen PT, Floyd M, Ford JM, Galvin M, Gerlach JL, Grotzfeld RM, Herrgard S, Insko DE (2005). A small molecule-kinase interaction map for clinical kinase inhibitors. Nature biotechnology.

[14] Vanhaesebroeck B, Khwaja A (2014). PI3Kdelta inhibition hits a sensitive spot in B cell malignancies. Cancer cell.

[15] Shanware NP, Bray K, Abraham RT (2013). The PI3K, metabolic, and autophagy networks: interactive partners in cellular health and disease. Annu Rev Pharmacol Toxicol.

