# Supplementary material for: Characterization of selective and potent PI3Kδ inhibitor (PI3KD-IN-015) for B-Cell malignances
Source: Oncotarget. 2016 Apr 12;7(22):32641–51. doi: 10.18632/oncotarget.8702 (PMC5078040; doi:10.18632/oncotarget.8702)
Supplement: Supplementary file 1 [file oncotarget-07-32641-s001.pdf]

Characterization of selective and potent PI3Kδ inhibitor (PI3KD-IN-015) for B-Cell malignances

SUPPLEMENTARY INFORMATION

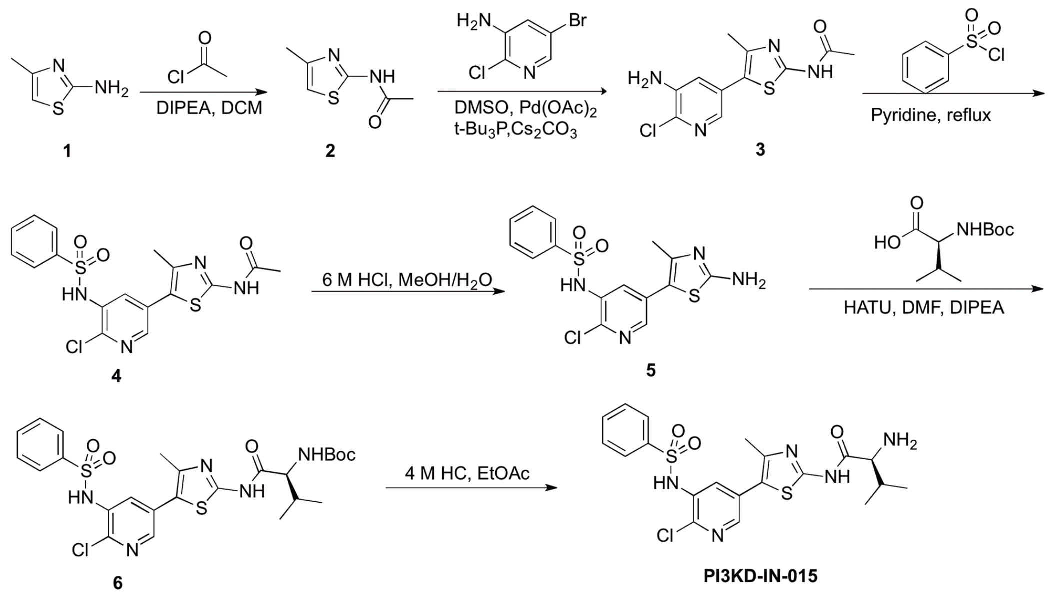

Chemical synthesis

All reagents and solvents were purchased from commercial sources and were used as received, unless specified otherwise, or prepared as described in the literature. All moisture-sensitive reactions were carried out using dry solvents under ultra pure argon protection. Glassware was dried in an oven at 140 °C for at least 12 h prior to use, and then assembled quickly while hot, sealed with rubber septa, and allowed to cool under a stream of argon. Reactions were stirred magnetically using Teflon-coated magnetic stirring bars. Commercially available disposable syringes were used for transferring the reagents and solvents. LC/MS were performed on an Agilent 6224 TOF using an ESI source coupled to an Agilent 1260 Infinity HPLC system operating in reverse mode with an Agilent XDB-C18 column (4.6×50 mm, 1.8 mm) using a water/acetonitrile (each with 0.2% (v/v) formic acid) gradient at a flow rate at 0.4 mL/min. ^{1}H and ^{13}C spectra were recorded on a Bruker 400 MHz NMR spectrometer. Chemical shifts are expressed in ppm. In the NMR tabulation, s indicates singlet; d, doublet; t, triplet; q, quartet; and m, multiplet. Flash column chromatography was conducted using silica gel (Silicycle 40–64 µm). The purities of all compounds were determined to be >95% by HPLC.

4-methylthiazol-2-amine(2)

To a solution of 2-amino-4-methylthiazole(15 g, 131.4 mmol, 1.00 equiv) in DCM (100 mL) was added TEA (24 mL, 144.5 mmol, 1.10 equiv) at 0 °C under argon. Then a solution of acetyl chloride (9.8 mL, 138.0 mmol, 1.05 equiv) in DCM (20 mL) was slowly added. The reaction mixture was stirred at 0 °C for 1h, then it was allowed to warm to room temperature for 14h. The resulting mixture was diluted with DCM (100 mL), washed with water (2×50 mL), brine (50 mL) and dried over anhydrous $MgSO_4$. Evaporation of the solvent afforded the crude product, which was crystallized from DCM to **2** as off a white solid.^{1}H NMR (400 MHz, DMSO-d_6) δ 12.04 (s, 1H), 6.71 (s, 1H), 2.25 (s, 3H), 2.13 (s, 3H).^{13}C NMR (101 MHz, DMSO-d_6) δ 168.63, 157.79, 146.96, 107.85, 22.93, 17.33. LC-MS (ESI, m /z):157.0342[M+H]$^+$.

N-(5-(5-amino-6-chloropyridin-3-yl)-4-methylthiazol-2-yl)acetamide(3)

To a solution of 3-amino-5-bromo-2-chloropyridine (8.3 g, 40.0 mmol, 1.00 equiv) in DMSO (80 mL) was added 2 (10.0 g, 64.0 mmol, 1.6 equiv) at room temperature under argon. Then CsF(21.5 g), $Pd(OAc)_2$ (1.03 g) and t-Bu_3P (20 mL, 0.2 M in heptane) was added. The reaction mixture was degassed

by argon. Then it was heated to 130 °C for 24h. The resulting mixture was cooled to room temperature and diluted with ice-cold water (1L). The precipitate was filtered and washed with water. The precipitate was dissolved in DCM/MeOH (1:1)(100 mL) and decolored by activated carbon. The yellow solid was diluted with diethyl ether (100 mL), and the yellow solid was filtered and dried to provide **3**.^{1}H NMR (400 MHz, DMSO-d_6) δ 7.68 (s, 1H), 7.25 (s, 1H), 5.75 (s, 2H), 2.35 (s, 3H), 2.15 (s, 3H).^{13}C NMR (101 MHz, DMSO-d_6) δ 169.28, 156.64, 143.65, 141.78, 135.28, 133.97, 129.06, 121.31, 120.01, 23.07, 16.64. TOF LC-MS (ESI, m/z): 283.0699[M+H]$^+$.

N-(5-(6-chloro-5-(phenylsulfonamido)pyridin-3-yl)-4-methylthiazol-2-yl)acetamide(4)

To a solution of 3 (2.50 g, 8.87 mmol, 1.00 equiv) in anhydrous pyridine (30 mL) was added benzensulfonyl chloride (1.14 g, 8.87 mmol, 1.00 equiv) at 0 °C under argon. Then it was allowed to warm to room temperature for 24h. The resulting mixture was concentrated to give the crude product, which was purified by flash chromatography as a white solid (eluting with MeOH in DCM 0-2%). ^{1}H NMR (400 MHz, DMSO-d_6) δ 12.30 (s, 1H), 10.54 (s, 1H), 8.35 (s, 1H), 7.80 (s, 2H), 7.69 (s, 2H), 7.63 (s, 2H), 2.30 (s, 3H), 2.19 (s, 3H).^{13}C NMR (101 MHz, DMSO-d_6) δ 169.18, 156.86, 145.66, 145.16, 144.03, 140.40, 134.32, 133.88, 131.24, 130.05, 129.02, 127.18, 118.63, 22.97, 16.46. LC-MS (ESI, m/z):423.0439[M+H]$^+$.

N-(5-(2-amino-4-methylthiazol-5-yl)-2-chloropyridin-3-yl)benzenesulfonamide(5)

To a solution of **4** (211 mg, 0.5 mmol, 1.00 equiv) in EtOH (4 mL) was added 6 N HCl (0.7 mL) at room temperature under argon. Then it was heated to reflux for 4h. The precipitate was cooled to room temperature and filtered to afford 5.^{1}H NMR (400 MHz, DMSO-d_6) δ 9.75 (s, 1H), 8.32 (s, 1H), 7.78 (d, *J* = 7.1 Hz, 2H), 7.69 (d, *J* = 6.7 Hz, 1H), 7.65 (s, 1H), 7.61 (d, *J* = 7.3 Hz, 2H), 2.21 (s, 3H).^{13}C NMR (101 MHz, DMSO-d_6) δ 168.27, 146.02, 145.36, 140.17, 134.71, 133.89, 131.30, 130.00, 127.28, 126.52, 111.42, 99.99, 13.11. TOF LC-MS (ESI, m/z):381.0490[M+H]$^+$.

(S)-tert-butyl1-(5-(6-chloro-5-(phenylsulfonamido)pyridin-3-yl)-4-methylthiazol-2-ylamino)-3-methyl-1-oxobutan-2-ylcarbamate (6)

To a solution of 5 (50 mg, 0.1 mmol, 1.00 equiv) in anhydrous DMF (2 mL) was added Boc-L-valine (33 mg, 0.15 mmol, 1.50 equiv), HATU (57 mg, 1.50 equiv) and DIPEA (0. 89 mL, 0.50 mmol, 5.0 equiv) at 0 °C under argon. The reaction mixture was stirred at 0 °C for 1 hour, then it was allowed to warm to room temperature for 20 hours. The resulting mixture was concentrated to dryness. The residue was diluted with water (30 mL), extracted with EtOAc(3×30 mL). The combined organic layers were washed with water(50 mL), brine (50 mL) and dried over anhydrous Na_2SO_4. Evaporation of the solvent provided the crude product, which was purified by flash chromatography (eluting with MeOH in DCM 0-1.5 %) to give the compound **6** as yellow solid (44 mg, yield 77%).^{1}H NMR (400 MHz, $CDCl_3$) δ 8.17 (d, *J* = 1.6 Hz, 1H), 8.02 (d, *J* = 1.8 Hz, 1H), 7.86 (d, *J* = 7.6 Hz, 2H), 7.63 (t, *J* = 7.5 Hz, 1H), 7.53 (t, *J* = 7.7 Hz, 2H), 4.31 (s, 1H), 2.39 (s, 3H), 2.33 (dd, *J* = 13.1, 6.6 Hz, 1H), 1.49 (s, 10H), 1.05 (d, *J* = 6.8 Hz, 3H), 1.00 (d, *J* = 6.8 Hz, 3H).^{13}C NMR (101 MHz, $CDCl_3$) δ 170.40, 155.94, 144.96, 144.44, 140.32, 138.52, 133.91, 130.54, 129.51, 129.13, 129.02, 127.22, 120.27, 38.74, 30.67, 28.45, 19.36, 17.87, 15.86. LC-MS (ESI, m/z):580.0699[M+H]$^+$.

(S)-2-amino-N-(5-(6-chloro-5-(phenylsulfonamido)pyridin-3-yl)-4-methylthiazol-2-yl)-3-methylbutanamide(7)

To a solution of 6 (22 mg, 0.038 mmol, 1.00 equiv) in EtOAc (1 mL) was added 1 mL of 4 M HCl (in EtOAc) at room temperature. The reaction mixture was stirred at room temperature for 1h. The resulting precipitate was filtered and washed with EtOAc to afford the **PI3KD-IN-015** as a white solid. ^{1}H NMR (400 MHz, CD_3OD) δ 8.34 – 8.20 (m, 1H), 8.07 – 7.97 (m, 1H), 7.84 (s, 2H), 7.67 (d, *J* = 3.2 Hz, 1H), 7.57 (d, *J* = 4.7 Hz, 2H), 4.03 (s, 1H), 2.42 (d, *J* = 2.9 Hz, 4H), 1.20 – 1.07 (m, 6H).^{13}C NMR (101 MHz, CD_3OD) δ 170.61, 166.02, 143.14, 142.64, 141.50, 138.41, 131.83, 131.68, 129.92, 127.50, 126.85, 125.42, 118.42, 57.14, 28.81, 16.03, 14.94, 12.96. LC-MS (ESI, m/z): 480.1137[M+H]$^+$.

SUPPLEMNTARY FIGURE AND TABLES

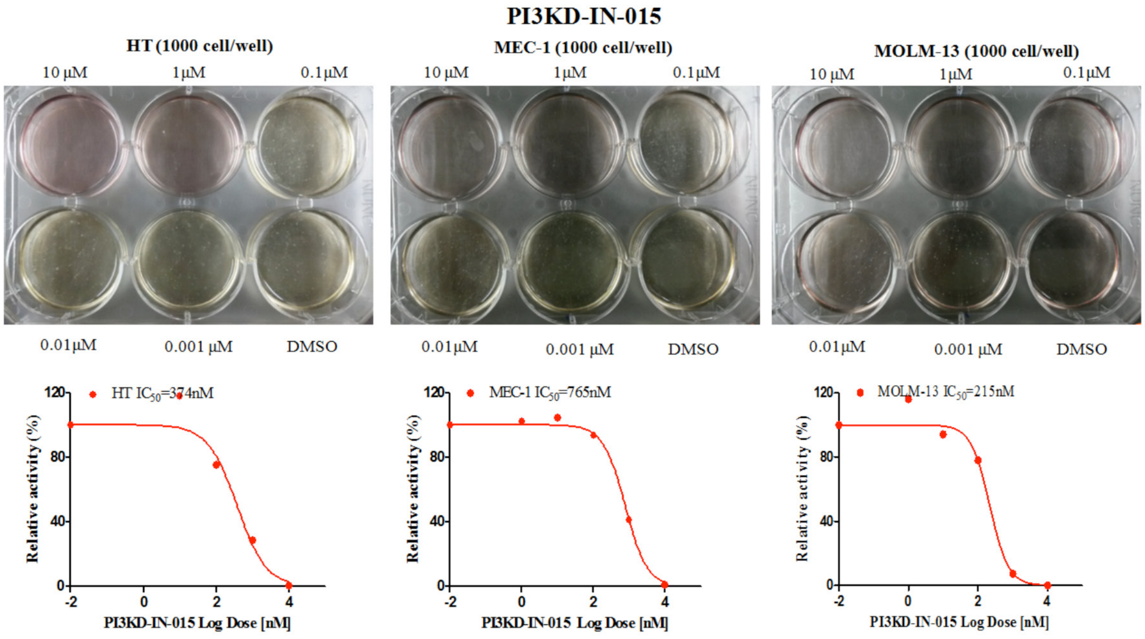

Supplementary Figure S1: Colony formation testing of PI3KD-IN-015 against HT, MEC, MOLM13 cells

Supplementary Table S1: KinomeScan of PI3KD-IN-015

(See Supplementary File 1)

Supplementary Table S2: Patient primary cell information

	Age	Gender	% Blasts	Pathology	Cytogenetics
AML1	72 (deceased)	M	77	AML	46,XY,del(11)(p11.2p13)[13]/46,Y,t(X;2)(q13;p24)[cp2]/46,XY,del(20)(q11.2)[2]/46,XY[cp3]
AML2	70	M	82	AML	46,XY,t(14;21)(q22;q22)[20].ish T(14;21)(AML1+;AML1+)[5/5]
AML3	36 (deceased)	M	92	AML	46,XY[20]
AML4	54	M	73	AML	46,XY[cp20]
AML5	55	M	Not available	AML	Not available
AML6	79	F	Not available	AML	Not available
